# Features of mHealth Apps for Tobacco Cessation Important to Black Adults: Discrete Choice Experiment

**DOI:** 10.2196/83919

**Published:** 2026-03-13

**Authors:** Chineme Enyioha, Lauren Gorstein, Sonia Clark, Adam O Goldstein, Roger Vilardaga, Lisa B Hightow-Weidman, Christine E Kistler

**Affiliations:** 1 Department of Family Medicine, University of North Carolina at Chapel Hill, 590 Manning Drive, Chapel Hill, NC, 27514, United States, 1 9842155048; 2 Lineberger Comprehensive Cancer Center, University of North Carolina at Chapel Hill, Chapel Hill, NC, United States; 3 Center for Health Promotion and Disease Prevention, University of North Carolina at Chapel Hill, Chapel Hill, NC, United States; 4 Department of Implementation Science, School of Medicine, Wake Forest University, Winston Salem, NC, United States; 5 College of Nursing, Florida State University, Tallahassee, FL, United States; 6 Division of Geriatric Medicine, University of Pittsburgh, Pittsburgh, PA, United States

**Keywords:** mobile health app, tobacco cessation, mHealth app preferred features, mobile health, cultural relevance, relatability, app preferences

## Abstract

**Background:**

Although mobile health (mHealth) apps for tobacco cessation augment traditional cessation methods and have contributed to increases in cessation rates, Black adults are underrepresented in mHealth app studies for tobacco cessation. As a result, their mHealth app preferences are not well-known.

**Objective:**

Our goal was to identify features of mHealth apps for cessation that are important to Black adults who use tobacco products.

**Methods:**

We developed an online discrete choice experiment with 12 pairs of hypothetical mHealth apps for tobacco cessation. Inclusion criteria included being 21 years or older, current use of any tobacco product, and identifying as Black or African American. Participants had to be interested in tobacco cessation and have a history of mHealth app use or be willing to use one in the future. From each pair of hypothetical apps within the survey, participants had to choose the app they preferred. Each hypothetical app was made up of 7 features developed from existing mHealth literature and prior qualitative work: graphics, marketing, strategies for quitting, connection with others, personalization, benefits of quitting, and health information. Each feature had up to 4‐5 levels (ie, variations of that attribute), and each hypothetical mHealth app was comprised of a random assortment of levels of features. Hierarchical Bayes estimation was used to determine the part-worth utility for each level within each feature for each participant, which was then used to calculate the importance score. Average importance scores across respondents were used to determine overall importance scores for each feature.

**Results:**

We had 901 adult participants. The mean age was 41 (SD 14.02) years, and about a third of participants (377/901, 42%) were female. Two-thirds of participants (549/901, 61%) had used an mHealth app in the past, and the great majority (786/901, 87%) indicated a willingness to use an app for health purposes in the future. The features had the following importance: graphics (16%), marketing (15%), strategies for quitting (15%), connection with others (14%), personalization (13%), benefits of quitting (13%), and health information (13%). Within features, strategies for quitting had the highest and third-highest levels of “making a step-by-step quit plan” and “recommendations to manage relapse or withdrawal,” respectively. Marketing had the second-highest level of “Historically Black Colleges and Universities–endorsed app.” Graphics had the fourth-highest level of “short video testimonials from people who successfully quit,” while connection with others had the fifth-highest level of “quit buddy program for support and accountability.”

**Conclusions:**

This study identified features of mHealth apps important to Black adult tobacco users. To enhance the appeal of mHealth apps to such adults, prioritizing inclusion of highly preferred levels in apps may lead to higher use and improved cessation.

## Introduction

Black adults in the United States who use tobacco products have a higher incidence of tobacco-related adverse health effects and mortality compared with adults from other racial or ethnic backgrounds [1,2]. Although Black adults who use tobacco attempt to quit and endorse intention to quit at a higher rate than White adults who use tobacco products, they are less likely to succeed [3]. One major reason for lower cessation rates for Black adults is less access to or likelihood to use evidence-based treatment, including lower rates of screening for smoking among Black patients and lower likelihood of getting cessation-related advice from health care professionals [4,5].

The use of mobile health (mHealth) interventions to support or augment mainstream treatment options has led to improved clinical outcomes for individuals with other chronic health conditions, including those from minority racial or ethnic groups [6,7]. mHealth interventions have contributed to success in tobacco cessation [8], often due to factors such as the ability to tailor content based on responses of the user, provision of real-time feedback, and low cost [9-11]. mHealth interventions for tobacco cessation can mitigate difficulties typically associated with more traditional interventions such as transportation issues, costs, and scheduling challenges [11].

Even with the known benefits of mHealth interventions for tobacco cessation, studies have shown that not all aspects of these interventions appeal to adults who use tobacco products, and participants frequently unsubscribe or disengage from an mHealth intervention program [12,13]. Black adults have more mobile phone use compared with White adults [14] but are underrepresented in mHealth studies [15]. Compounding their lack of representation, their preferences for features of tobacco-related mHealth apps have not been well documented [16,17]. One study of a single mHealth app found that Black adults who smoke cigarettes prefer unique features of mHealth apps, such as information on benefits and strategies for quitting, video testimonials, and content relevant to Black adults who use tobacco products to enhance inclusivity [18]. Findings from this study were based on a single mHealth app, which may have limited the number of features that participants were exposed to. Consequently, there remains a gap in the evidence about features of mHealth apps for tobacco cessation that may appeal to Black adults who use tobacco products. Research to remedy this gap may increase use and the overall success rate of tobacco cessation for this population.

A discrete choice experiment (DCE) is a rigorous preference elicitation method [19], developed in behavioral economics to identify which features of a product are important to consumer purchases [20]. In DCEs, respondents view hypothetical products or services with different features and choose ones they prefer, which helps to identify exact components or characteristics that make a product more appealing [21]. DCEs help to determine the relative importance of individual features of a product or intervention relative to other features and identify trade-offs between features that respondents may accept [22]. Compared with other methods to evaluate preferences such as ratings, ranking questions, or qualitative designs, DCEs have better performance with regard to internal consistency and validity [23]. DCEs have been used in some areas of tobacco research, including preferences for cigarette packaging design, pharmacotherapy, and features of electronic cigarettes [24,25]. Little research has examined the application of DCEs to mHealth interventions for the reduction or cessation of tobacco use. The objective of this study was to use a DCE experiment to identify which features of mHealth apps for tobacco cessation are important to Black adults who use tobacco products.

## Methods

### Study Design and Participant Recruitment

We conducted a 1-time DCE, with a survey consisting of 12 choice tasks where participants were asked to choose between 2 hypothetical mHealth apps, and each app was made up of a combination of different features. Participants were recruited from Qualtrics, a validated source for online recruitment of participants for surveys with reliable data in a short period of time [26]. Qualtrics applies a comprehensive system to recruit from third-party panels, email lists, as well as social media, and participants are targeted for recruitment based on their panel profile information. Quotas for sex and age were provided, and eligibility criteria included ages 21 years or older, who were identified as Black or African American, and who currently used cigarettes or other tobacco products. Current use of tobacco products was defined as either having smoked at least 100 cigarettes in their lifetime and currently smoking in the past 30 days and/or past 30-day use of another tobacco product. Participants had to reside in the United States, complete the survey in English, report interest in tobacco cessation, and have a history of using an mHealth intervention program in the past or willingness to use one in the future. Respondents had to complete a screening survey to confirm eligibility and pass through a CAPTCHA test after receiving the study invitation from Qualtrics to prevent automated bots. Qualtrics also applied verification methods such as IP address checks to confirm the authenticity of participants in addition to a system that detects duplicate responses and evaluates for fraud risk based on specific device and browser attributes. Consent to participate in the study was obtained through the Qualtrics opt-in system. Through this system, participants have to opt in to participate in any research study. An information sheet with a description of the experiment and the rights of participants was included at the beginning of the experiment.

The study was also guided by the CHERRIES (Checklist for Reporting Results of Internet E-Surveys; Checklist 1) [27].

### Sample Size

The sample size was estimated based on power calculations using the number of features and levels [28]. In DCEs, statistical power depends on factors such as the sample size, number of choice sets, alternatives within each set, and expected effect size or β coefficients for the DCE model [28]. Based on these parameters, it was determined that a sample size of 900 was needed to achieve over 80% power to detect meaningful differences between features and levels at 95% confidence level.

To ensure the quality of data collected, the team applied predefined criteria related to speeding or incompletion. We defined speeders as participants who completed the survey in less than half the median duration of time from start to completion for the whole sample of participants. Because Qualtrics also has an internal system to automatically remove duplicates, we are only able to report exclusions that were identified after a manual check of the dataset.

### Survey Design

We developed a comprehensive list of mHealth app features for tobacco cessation from our formative work and the literature, and then conducted focus groups to improve the list. We decided not to include some features from the comprehensive list in the DCE because those features were either unanimously endorsed as favorable, for example, some form of reward or incentive, or they are basic or core features that need to be included in mHealth apps, for example, the option to anonymize an individual’s identity. The experiment was focused on features with unclear or variable levels of appeal. The final list included the following potential mHealth features*:* benefits of quitting, strategies for quitting, graphics, personalization, health information, marketing, and connection with others (Table 1).

**Table 1.** Final mobile health app features and definitions.

Feature	Definition	Levels
Graphics	Types of images that an app can have such as images about the health effects of smoking and images of people giving testimonials about their cessation journey. These images could be real human images or animation images representing one or several races.	Short video testimonials of people who successfully quit. Photos that feature Black people who successfully quit. Written testimonials from people who successfully quit. Photos of real people with health issues from tobacco use. Photos that feature racially diverse people who have successfully quit.
Marketing	Information about endorsement of the app by individuals or groups as well as inclusion of music and pop culture references in the app.	Features culturally relevant music and pop culture references. Features different types of music and pop culture references. Is endorsed by a Black celebrity who successfully quit tobacco products. Is endorsed by a celebrity who successfully quits tobacco products. Is endorsed by a historically Black college or university that this app can help people quit.
Strategies for quitting	Information about how to quit smoking such as other smoking cessation methods like the patch, gum, and medication. It can also include information about how to deal with stress related to quitting and how to deal with distractions.	Makes a step-by-step quit plan. Recommends strategies for relapse or withdrawal symptoms. Links to online resources for tobacco cessation. Recommends tobacco cessation medications to help with quitting. Interactive games and videos to distract from smoking.
Connection with others	Ability to interact with other people outside of the app. This may be other users of the app, other smokers, interacting with health care personnel or a quit expert.	Provides the ability for you to chat with other users within the app via a message board. Provides the option to interact with a variety of support professionals (eg, quit coach). Contains links to support groups on Facebook or motivational Instagram pages from the app. Has a leaderboard that ranks your progress toward your cessation goals compared to other app users. Has a quit buddy program within the app to help you support others and keep you accountable.
Personalization	Ability for a user to tailor or adjust components of the app based on their preferences. For example, a user may be able to upload personal pictures or motivation images or songs to the app.	Has the ability for you to keep a journal in the app. Includes the option to create a vision board to help you visualize your life without tobacco products. Includes the option to upload your personal goals and motivations for quitting. Includes the option to upload motivational videos or music of your choice. Has the ability to set reminders and notifications.
Benefits of quitting	Information about what an individual who smokes can gain from quitting. These can be health benefits, financial benefits, or aesthetic benefits.	How much money saved since quitting. Lowers the risk of a heart attack. Lowers the chances of getting cancer. Improves physical appearance.
Health information	Information about the way tobacco products have been advertised and the health consequences of using tobacco products.	Contains information about how people who use tobacco products have a higher risk of blood pressure and heart problems. Contains information about how Black people who use tobacco products have a higher risk of blood pressure and heart problems. Has information about the historical targeting of the Black community by tobacco companies. Has information about how menthol is added to tobacco products to make them highly addictive.

Following the International Society for Pharmacoeconomic Outcomes Research for Good Research Practice in Health guidelines [29], we designed a conjoint DCE with 12 choice tasks using Sawtooth Software (Sawtooth Software, Inc) [30]. We used design principles of orthogonality and level balance to ensure even distribution of levels such that levels had a similar number of appearances in the experiment and could be selected without dependence on other levels or features [31]. Each choice task was made up of a pair of hypothetical mHealth apps for tobacco cessation containing 1 randomly assigned level from each of the 7 features. We applied the balanced overlap design method in generating the DCE tasks, which allowed for simple overlap of levels within features for each task. This approach reduces excessive and unnecessary processing of information and enables respondents to focus on features that differ, which increases the likelihood of making more meaningful trade-offs as they complete each task [31]. To further reduce cognitive burden and improve consistency of choice, we applied color coding. That is, if both apps shown contained the same level of a feature, that level was grayed out so participants could focus on the differences between the 2 apps [32]. To confirm the quality of the design, Sawtooth’s test design function was applied to check the integrity of the design with simulated data and verify that the relevant effects can be estimated [31]. We conducted cognitive interviews with 6 participants in an iterative manner to ensure clarity of the questions asked in the DCE. After reaching saturation, we carried out a pilot test of the survey with 53 participants recruited from Qualtrics to examine for illogical response patterns. After all concerns were addressed, we fielded the final version of the survey from April to July 2024. Each Qualtrics participant was assigned a unique identifier based on their IP addresses. Participants were compensated in accordance with the set compensation rate at Qualtrics for survey completion.

### Measures

The first part of the survey began with general questions about demographics, tobacco use history, and health status. Participants were also asked questions about their age, sex, race, and ethnicity since we included those who identified as Black plus another race. We also collected information on health status, state of residence, level of education, and household income.

The second part of the survey contained an explanation of each mHealth app feature, and each feature had 4‐5 levels (Table 1). An example of a DCE using car features was presented. After the example, participants were presented with the following scenario for each choice task: “Imagine that two new mHealth apps for tobacco cessation were recently developed and you want to quit using tobacco products. If these two apps were your only two options, which app would you choose? App A or App B?” Table 2 illustrates an example of one of the 12 DCE tasks that participants completed.

**Table 2.** Example of a discrete choice task.

Feature	App A	App B
Benefits of quitting tobacco products	Has information about how quitting tobacco products can lower your risk of a heart attack	Includes information about how quitting tobacco products can lower your chances of getting cancer
Strategies for quitting	Has recommendations about how to manage relapse or symptoms of withdrawal	Has recommendations about how to manage relapse or symptoms of withdrawal
Graphics	Contains short video testimonials from people who successfully quit	Contains written testimonials from people who successfully quit
Personalization	Has the ability to set reminders and notifications	Has the ability to set reminders and notifications
Health information	Contains information about how people who use tobacco products have a higher risk of blood pressure and heart problems	Has information about how menthol is added to tobacco products to make them highly addictive
Marketing	Features different types of music and pop culture references	Is endorsed by a Black celebrity who successfully quit tobacco products
Connection with others	Provides the option to interact with a variety of support professionals (eg, a quit coach)	Provides the option to interact with a variety of support professionals (eg, a quit coach)
	Select	Select

### Statistical Analysis

Data for demographics, tobacco use, health status, and other participant characteristics were analyzed using descriptive statistics (SPSS Statistics, version 29.0.1.0; IBM Corp). Differences among age groups were assessed using 1-way ANOVA, with statistical significance set at *P*<.05.

The response data from the DCE were analyzed with the help of Sawtooth Analytics. We first determined part-worth utilities at the individual level with hierarchical Bayes estimation within Sawtooth Software [31]. Part-worth utilities indicate the preference for levels within each feature. More preferred levels have higher utilities (above 0), and dispreferred levels have the lowest utilities (below 0). With hierarchical Bayes, we were able to apply a Bayesian method to fit a multinomial logit model at the individual level, which allows for heterogeneity while strengthening the overall sample. Our calculations were based on 12 choice tasks per participant, with each task having 2 alternatives.

Next, we determined the importance score for each feature at the individual level by calculating the range of part-worth utilities for levels within the specific feature and dividing by the sum of ranges across all features multiplied by 100. The importance score is the percentage of choice due to each feature or the measure of influence of a feature on the decision-making process to choose a product. The higher the importance score, the higher the influence of that feature on the choice of the product, in this case, the mHealth app. For each feature, the importance score at the individual level was averaged across all participants to obtain the overall importance score. We summarized aggregate preferences with multinomial logit regression with effects coding, with sample-level coefficient and goodness-of-fit statistics provided to enable us to obtain both disaggregate utilities and aggregate estimates.

In addition, we conducted exploratory analysis with paired 2-tailed *t* tests to compare importance scores across features and to compare part-worth utilities of levels within features in all possible combinations to determine statistical significance.

### Ethical Considerations

The hypotheses, study design, and analytical plan were preregistered on AsPredicted [33] (#165678), and the study was reviewed by the institutional review board of the University of North Carolina at Chapel Hill and exempted (Study # 23‐2131). At the beginning of the experiment, we included an information page with the study description and the rights of participants. All participants provided informed consent through the Qualtrics opt-in system before participating in the study. Participants were compensated according to Qualtrics set rate for compensation after completion of surveys.

## Results

### Overview

The analytical sample included 901 respondents. Of the initial 1304 participants, 369 were excluded due to speeding, that is, those who spent less than half median duration of time spent by all participants (239 seconds) to complete the survey, and 34 were excluded due to incomplete responses. The mean age of participants was 41 (SD 14.02) years, and 42% (377/901) were female participants. Most participants (845/901, 94%) were non-Hispanic Black and had annual household income above the federal poverty level (726/901, 81%). In total, 61% (549/901) of participants reported that they had used an mHealth app in the past, and 87% (786/901) had willingness to use an mHealth app in the future. In total, 82% (738/901) of participants reported current cigarette use, 79% (714/901) reported use of other tobacco products besides tobacco cigarettes, and 82% (738/901) reported a history of a prior quit attempt (Table 3).

**Table 3.** Demographics, mobile health app use, and tobacco use characteristics.

	Total	21‐24	25‐49	50+	*P* value
Age (years)					
Mean (SD)	41.15 (14.02)	22.71 (1.12)	36.74 (6.81)	60.93 (8.33)	<.001
Race, n (%)					
Black or African American	879 (98)	100 (95)	557 (97)	222 (99)	.19
Black or African American and some other race or origin	22 (2)	5 (5)	14 (2)	3 (1)	.19
Non-Hispanic	845 (94)	97 (92)	530 (93)	218 (97)	.09
Sex, n (%)					.74
Male	524 (58)	58 (55)	337 (59)	129 (57)	
Female	377 (42)	47 (45)	234 (41)	96 (43)	
Education, n (%)					.03
High school or less	296 (33)	37 (35)	176 (31)	83 (37)	
Some college	244 (27)	23 (22)	154 (27)	67 (30)	
Associate	132 (15)	19 (18)	77 (14)	36 (16)	
Bachelor	176 (20)	18 (17)	123 (22)	35 (16)	
Graduate degree or higher	49 (5)	8 (8)	37 (6)	4 (2)	
Annual household income, n (%)					
Above federal poverty guidelines	726 (81)	82 (78)	465 (81)	179 (80)	.66
App use, n (%)					
Yes, I have used an app	549 (61)	76 (78)	377 (72)	96 (50)	<.001
Willingness to use app, n (%)					<.001
Yes	786 (87)	92 (88)	522 (91)	172 (76)	
Not sure	115 (13)	13 (12)	49 (9)	53 (24)	
Tobacco use, n (%)					
Yes	738 (82)	75 (71)	488 (85)	175 (78)	<.001
Current cigarette use in past 30 days, n (%)					.01
Less than 10 days	111 (15)	17 (23)	59 (12)	35 (20)	
10 through 20 days	137 (19)	20 (27)	101 (21)	16 (9)	
Greater than 20 days	489 (66)	38 (51)	327 (67)	124 (71)	
Current other tobacco product use, Yes, n (%)					
Cigars, for example, little cigars, cigarillos, or large cigars	536 (59)	74 (71)	400 (70)	62 (28)	<.001
Smokeless tobacco, for example, chewing tobacco, snuff, dip, or snus	34 (4)	6 (6)	25 (4)	3 (1)	.09
e-Cigarettes or other vaping devices	95 (10)	10 (9)	55 (10)	30 (13)	.29
Waterpipe tobacco or hookah	2 (0)	0 (0)	1 (0)	1 (0)	.80
Other types of oral nicotine products, such as Velo lozenges, Rogue tablets, Lucy gum, or Pixotine	1 (0)	0 (0)	1 (0)	0 (0)	.99
Other	31 (3)	2 (2)	9 (2)	20 (9)	<.001
None	187 (21)	10 (10)	75 (13)	102 (45)	<.001
Time to first smoke after waking, n (%)					.75
Less than 5 minutes	225 (25)	28 (27)	134 (24)	63 (28)	
6‐30 minutes	521 (58)	62 (59)	347 (61)	112 (50)	
61 or more minutes	154 (17)	15 (14)	90 (16)	49 (22)	
Ever tried to quit, n (%)					.66
Yes, and I was successful	232 (26)	31 (30)	140 (25)	61 (27)	
Yes, and I was not successful	506 (56)	55 (52)	322 (57)	129 (57)	
Quit attempts in the last 12 months, n (%)					.15
Yes	405 (45)	56 (53)	246 (43)	103 (46)	

### Overall Importance of Features of mHealth App for Tobacco Cessation

Of all 7 mHealth app features, graphics was determined to have the highest average importance score at 16.5% (95% CI 16.2-16.9). Marketing and strategies for quitting were next with importance scores of 15.1% (95% CI 14.7-15.4) and 15% (95% CI 14.7-15.4), respectively. Connection with others had an importance score of 14.3% (95% CI 14.0-14.6), while personalization was fifth with an importance score of 13.4% (95% CI 13.1-13.7). The lowest-rated features were benefits of quitting and health information with importance scores of 13.1% (95% CI 12.8-13.5) and 12.6% (95% CI 12.2-12.9), respectively. Paired 2-tailed *t* test revealed that importance scores were statistically significantly different when compared with one another (*P*<.001) except for comparison between marketing and strategies for quitting (*P*=.46) and between personalization and benefits of quitting (*P*=.19). We found no statistically significant differences in the analysis by age group and sex.

### Part-Worth Utility or Preferences for Levels Within Features

Within each feature, some levels were preferred more than others. The 5 most preferred levels were found within strategies for quitting, marketing, graphics, and connection with others. Within features, strategies for quitting had the highest and third-highest levels of “making a step-by-step quit plan” with a part-worth utility of 21.03 (95% CI 18.36-23.70) and “recommendations to manage relapse or withdrawal” with a part-worth utility of 16.70 (95% CI 14.68-18.73), respectively. Marketing had the second-highest level of “Historically Black Colleges and Universities–endorsed app” with a part-worth utility score of 18.33 (95% CI 15.70-20.95). Graphics had the fourth-highest level of “short video testimonials from people who successfully quit” with a part-worth utility score of 15.55 (95% CI 13.12-17.98), while connection with others had the fifth-highest level of “quit buddy program for support and accountability” with a part-worth utility score of 14.61 (95% CI 12.27-16.95; Table 4).

**Table 4.** Part worth utility scores of levels within features.

Features and levels		Part worth utility, mean (95% CI)
Graphics		
	Contains short video testimonials from people who successfully quit.	15.55 (13.12 to 17.98)
	Has photos that feature Black people who have successfully quit.	0.93 (−2.24 to 4.11)
	Contains written testimonials from people who successfully quit.	−1.92 (−4.47 to 0.64)
	Has photos of real people with health issues from tobacco use (eg, lung or throat cancer).	−2.31 (−5.39 to 0.78)
	Has photos that feature people from a variety of races who have successfully quit.	−12.26 (−14.71 to −9.80)
Marketing		
	Is endorsed by a historically Black college or university that this app can help people quit.	18.33 (15.70 to 20.95)
	Is endorsed by a Black celebrity who successfully quit tobacco products.	5.89 (3.18 to 8.61)
	Features culturally relevant music and pop culture references.	−5.69 (−8.03 to −3.36)
	Features different types of music and pop culture references.	−5.73 (−8.34 to −3.12)
	Is endorsed by a celebrity who successfully quits tobacco products.	−12.80 (−14.97 to −10.62)
Strategies for quitting		
	Helps you make a step-by-step quit plan.	21.03 (18.36 to 23.70)
	Has recommendations about how to manage relapse or symptoms of withdrawal.	16.70 (14.68 to 18.73)
	Contains links to informational websites and free resources for tobacco cessation.	−10.62 (−12.83 to −8.41)
	Includes information about recommended tobacco cessation medications that can help with quitting.	−11.96 (−14.33 to −9.59)
	Has interactive games and videos to distract you from smoking and provide other forms of entertainment.	−15.16 (−17.74 to −12.58)
Connection with others		
	Has a quit buddy program within the app to help you support others and keep you accountable.	14.61 (12.27 to 16.95)
	Provides the option to interact with a variety of support professionals (eg, quit coach).	11.98 (9.56 to 14.40)
	Has a leaderboard that ranks your progress toward your cessation goals compared to other app users.	−0.09 (−2.39 to 2.20)
	Provides the ability for you to chat with other users within the app via a message board.	−9.40 (−11.85 to −6.94)
	Contains links to support groups on Facebook or motivational Instagram pages from the app.	−17.10 (−19.23 to −14.97)
Personalization		
	Includes the option to create a vision board to help you visualize your life without tobacco products.	5.92 (4.02 to 7.83)
	Has the ability to set reminders and notifications.	5.60 (3.11 to 8.10)
	Includes the option to upload your personal goals and motivations for quitting.	2.97 (0.52 to 5.43)
	Has the ability for you to keep a journal in the app.	−2.88 (−4.95 to −0.82)
	Includes the option to upload motivational videos or music of your choice.	−11.62 (−13.96 to −9.28)
Benefits of quitting		
	Has information about how much money you can save by quitting tobacco products.	8.19 (5.78 to 10.61)
	Has information about how quitting tobacco products can lower your risk of a heart attack.	4.38 (1.98 to 6.79)
	Includes information about how quitting tobacco products can lower your chances of getting cancer.	−4.28 (−6.69 to −1.86)
	Includes information about how quitting tobacco products can improve physical appearance.	−8.30 (−10.73 to −5.87)
Health information		
	Contains information about how Black people who use tobacco products have a higher risk of blood pressure and heart problems.	9.40 (6.68 to 12.12)
	Contains information about how people who use tobacco products have a higher risk of blood pressure and heart problems.	9.40 (6.68 to 12.12)
	Has information about the historical targeting of the Black community by tobacco companies.	−6.22 (−8.21 to −4.23)
	Has information about how menthol is added to tobacco products to make them highly addictive.	−8.44 (−10.87 to −6.01)

Least preferred levels were also found. The top 5 dispreferred levels were found within connection with others, strategies for quitting, marketing, and graphics. Within features, connection with others had the least preferred level of “links to support groups on social media” with a part-worth utility of −17.10 (91% CI −19.23 to −14.97), while strategies for quitting had the second and fifth least preferred levels of “interactive games and videos to distract from smoking” with a part-worth utility score of −15.16 (95% CI −17.74 to −12.58) and “recommended tobacco cessation medications to help with quitting” with a part-worth utility of −11.96 (95% CI −14.33 to −9.59). Marketing had the third least preferred level of “endorsement by a celebrity who successfully quit” with a part-worth utility of −12.80 (95% CI −14.97 to −10.62), while graphics had the fourth dispreferred utility of “ photos of racially diverse people who successfully quit” with a part-worth utility of −12.26 (95% CI −14.71 to −9.80; Table 4).

## Discussion

### Principal Findings

This study identified preferred features of mHealth apps for tobacco cessation that appeal to Black adults who use tobacco products. While all features had some importance, graphics, marketing, strategies for quitting, and connection with others were the most important. We also found that within each feature, some levels were highly preferred, while others were highly dispreferred. To enhance the appeal of mHealth apps to Black adults who use tobacco products, prioritizing the inclusion of highly preferred levels and features in apps may lead to higher use and improved cessation.

Graphics was the most important mHealth feature, particularly video testimonials from others who successfully quit using tobacco. Dynamic graphics such as video testimonials are known to enhance use and interest in mHealth apps for several health conditions [18,34]. One study focused on developing a culturally relevant app for Black individuals on cardiovascular health showed that participants were interested in video narratives from prior participants of a program [34]. Video testimonials can improve the relatability and relevance of the app for the target population [34,35], which can increase engagement and use. Another study to examine features that Black adults prefer in tobacco cessation mHealth apps based on one app found that providing content through videos was appealing [18]. Using videos as a means to provide content has also been associated with increased retention of information [36].

In addition, within the levels of the graphics feature*,* photos of people from diverse races who successfully quit were significantly dispreferred. The evidence regarding preferences for racially diverse features in mHealth apps is mixed. While our findings support the importance of inclusivity with mHealth app features [37], a concern also exists for targeting, mentioned previously in the literature [18]. The significant dispreference observed may be reflective of the desire or need to protect one’s self from interventions that may be considered racially targeted or that could potentially reinforce negative stereotypes, a concern highlighted in studies of medical mistrust [18,38-40]. Another factor that may have contributed to this finding is the static nature of this type of graphic. Images presented in a dynamic mode such as videos tend to be more appealing, while a lack of appeal and low likelihood of engagement with static information in mHealth apps has been described in the literature [35].

Marketing for the app was also found to be important, especially endorsement by a reliable source such as Historically Black Colleges and Universities (which was the most important level) or a Black celebrity. Relevant health information is valued by app users [37], more so if the information is from a reliable source [34]. Having a trusted group or individual endorse the app increases credibility and trust [41], which in turn can increase the likelihood of use of the app. Accuracy of information and intention toward end users are major concerns that tend to influence the decision-making process of target users [42]. As demonstrated in previous research, these concerns are especially relevant in light of the longstanding challenges associated with medical distrust [43,44]. Many Black adults who use tobacco products may be less likely to trust an mHealth app that lacks culturally relevant features and is promoted through mainstream channels, including non-Black celebrity endorsements.

Strategies for quitting were equally important, and similar to our study, previous research has shown that this feature is very important to other mHealth app users [18,45]. Provision of a practical guide for quitting has been determined to be highly desired in mHealth apps in general [45], which was the most important level of this feature in our study. One study found that Black adults who use tobacco products prefer strategies for quitting in an mHealth app, including ways to deal with cravings and healthy ways to deal with stress other than resorting to tobacco [18]. The next most important feature levels were a step-by-step plan and information on how to deal with challenges such as relapse or withdrawal symptoms. In that study, participants expressed interest in learning about alternatives to quitting abruptly (ie, the “cold turkey” method) and were particularly receptive to combining the app with pharmacotherapy [18].

The limited interest in pharmacotherapy and tobacco cessation resources observed in this study is not unexpected, as Black adults are less likely to be screened for tobacco use and, consequently, less likely to engage in discussions about cessation options [46]. Although dispreferred, the provision of this information within an mHealth app can be a key step to reducing failure rate and increasing the chances of sustained success with cessation for the target population. While interactive games in mHealth apps have been linked to increased engagement and enhanced self-efficacy [47,48], other studies, including our own, have found that such features may be less influential or valued by users [49,50].

We also found that connecting with other app users or health professionals through the app was important to participants, and such connections have been shown to keep users more engaged with apps [34,37,42]. Previous research shows that the addition of a program within the app for users to meaningfully compete among themselves is an attractive component of an app [35,37]. Participants can use competition as a way to challenge or motivate themselves as a way to reach their health goals. Similarly, connection with a health professional through the app can further strengthen the intervention [51], especially if such a connection can mitigate the need to have an in-person encounter with a provider for tobacco cessation concerns. Participants showed little interest in interacting with other users beyond accountability purposes or in connecting through social media groups. This aligns with other studies reporting the limited appeal of social networking features within mHealth apps [42,45]. Although some users may be open to sharing their health information, maintaining privacy, especially from friends on social media, may help reduce feelings of vulnerability often associated with efforts to improve one’s health [42,52].

Consistent with our findings, mHealth apps that offer personalization features, such as goal setting, reminders, and notifications, are well-established as desirable and beneficial to users [34,52]. Features that keep users on track about their smoking-related behavior [18,37], provide a self-management or monitoring system [53], and enhance the ability for users to monitor their own progress have been associated with a higher likelihood of achieving the target health outcome [51,53]. Tracking positive outcomes, such as money saved by not purchasing tobacco products or the number of days between use, has also been shown to be a helpful feature for mHealth app users [18]. In addition, notifications or reminders that help individuals focus on their reasons for quitting can help to keep users motivated and more engaged with the mHealth app. Allowing users to determine the timing and frequency of these reminders can also help to further enhance the appeal and use of the app [42].

Information about the benefits of quitting was the second to the lowest importance score overall, but within that feature, participants preferred information about the financial benefits of quitting. Similar to our study, the benefit of saving money by quitting has been mentioned as appealing content for mHealth apps for tobacco cessation [35,45,54]. It is well established that many individuals who use tobacco products also experience financial hardship [55,56]. Tobacco cessation can positively impact cash flow by reducing the ongoing costs associated with tobacco use. For example, in 2025, the average cost for a pack of cigarettes in the United States is about US $10 [57]. Including information in an mHealth app about the potential monthly or annual savings from quitting tobacco may motivate users to stay engaged in the cessation process.

Within the benefits of quitting, participants also preferred information about the reduced cardiovascular risk associated with cessation but dispreferred information about reduced risk of cancer or the aesthetic benefits of quitting. Information about the association between tobacco use and an adverse health effect like cancer has been well established, and it is possible that participants were already well informed about the cancer risk; hence, they did not think that more information would be beneficial. Other studies also show that information about the harms of smoking in an mHealth app may not be as appealing [45].

Health information was the least important feature overall; however, participants expressed specific interest in learning about the risks of adverse cardiovascular outcomes, particularly as they relate to Black adults who use tobacco products. Studies have shown that presenting relevant health information can generate interest because the information is more relatable [18]. Information about the addition of menthol to tobacco products and its role in addiction was not appealing, as well as information about how the tobacco industry has targeted Black communities. Although information about the tobacco industry targeting can be effective by evoking negative emotions toward the industry [58], the negative response observed in this study may reflect broader concerns about racial targeting and generalized mistrust. Discomfort with targeted interventions among Black adults has been well documented in previous research [43,59,60]. While presenting health information can be beneficial, it should be delivered in a way that is inclusive and does not inadvertently isolate Black adults from broader user groups.

### Limitations

We used a convenience sample, and the majority of participants reported household incomes above the federal poverty level and had attained a college degree or higher. As a result, the findings may not be generalizable to broader populations. Additionally, only a small proportion of respondents were older adults, who may be less comfortable with technology [52]. For analysis, individuals who identified as multiracial (Black and another race) were grouped with Black participants. Although multiracial participants represented only 2% of the study sample, combining them with Black participants may have affected our findings. We also excluded speeders and participants with incomplete or duplicate responses from our analysis. Although the affected participant made up less than 1% of the total sample, this may have disproportionally affected certain subgroups of participants. In addition, although we conducted several paired 2-tailed *t* tests to check for differences in importance scores and utilities across features and levels, we did not formally control for multiple comparisons; hence, some significant differences found may be due to chance. These analyses were exploratory, and our primary conclusions were based on the hierarchical Bayes estimations. Our findings are limited to the specific features and levels included in the DCE, which do not represent an exhaustive or comprehensive list. While we selected features based on existing literature and our qualitative work, it is possible that other features, equally or even more appealing to users, were not captured. Finally, we cannot determine whether incorporating all preferred features into an mHealth app will lead to sustained and meaningful use, since DCEs only capture stated preferences. Further research is needed to assess whether an app designed with these features ultimately improves tobacco cessation rates.

### Conclusions

In summary, the role of mHealth apps for tobacco cessation is promising. This study highlights features of mHealth apps for tobacco cessation that highly appeal to Black adults who use tobacco products. Inclusion of these highly preferred features: graphics, marketing, strategies for quitting, and connection with others in a mHealth app, and more importantly, preferred levels within these features should be considered in the design and development process. The inclusion of preferred features may increase the appeal and use of the app by the target population. Although end-user preferences are critical in mHealth app development, some less preferred features may still offer meaningful benefits for tobacco cessation and should be carefully reconsidered before being excluded entirely. Future research to test the effectiveness of an app with these preferred features is needed.

## Supplementary material

10.2196/83919 Checklist 1 CHERRIES checklist.

## Abbreviations

CHERRIES: Checklist for Reporting Results of Internet E-Surveys
DCE: discrete choice experiment
mHealth: mobile health

## References

[1] Ho JY, Elo IT (2013). The contribution of smoking to Black-White differences in U.S. mortality. Demography.

[2] Roberts ME, Colby SM, Lu B, Ferketich AK (2016). Understanding tobacco use onset among African Americans. Nicotine Tob Res.

[3] Ranney LM, Baker HM, Jefferson D, Goldstein AO (2016). Identifying outcomes and gaps impacting tobacco control and prevention in African American communities. J Health Dispar Res Prac.

[4] Pampel FC (2008). Racial convergence in cigarette use from adolescence to the mid-thirties. J Health Soc Behav.

[5] Bates-Pappas GE, Schofield E, Chichester LAR (2024). Universal tobacco screening and opt-out treatment referral strategy among patients diagnosed with cancer by race and ethnicity. JAMA Netw Open.

[6] Philis-Tsimikas A, Fortmann AL, Godino JG (2022). Dulce Digital-Me: protocol for a randomized controlled trial of an adaptive mHealth intervention for underserved Hispanics with diabetes. Trials.

[7] Enyioha C, Hall M, Voisin C, Jonas D (2022). Effectiveness of mobile phone and web-based interventions for diabetes and obesity among African American and Hispanic adults in the United States: systematic review. JMIR Public Health Surveill.

[8] Bricker JB, Watson NL, Mull KE, Sullivan BM, Heffner JL (2020). Efficacy of smartphone applications for smoking cessation: a randomized clinical trial. JAMA Intern Med.

[9] Chu KH, Matheny SJ, Escobar-Viera CG, Wessel C, Notier AE, Davis EM (2021). Smartphone health apps for tobacco cessation: a systematic review. Addict Behav.

[10] Battalio SL, Pfammatter AF, Kershaw KN, Hernandez A, Conroy DE, Spring B (2022). Mobile health tobacco cessation interventions to promote health equity: current perspectives. Front Digit Health.

[11] Whittaker R, McRobbie H, Bullen C, Rodgers A, Gu Y (2016). Mobile phone-based interventions for smoking cessation. Cochrane Database Syst Rev.

[12] Müssener U, Bendtsen M, McCambridge J, Bendtsen P (2016). User satisfaction with the structure and content of the NEXit intervention, a text messaging-based smoking cessation programme. BMC Public Health.

[13] Abroms LC, Boal AL, Simmens SJ, Mendel JA, Windsor RA (2014). A randomized trial of Text2Quit: a text messaging program for smoking cessation. Am J Prev Med.

[14] Chui M, Gregg B, Kohli S, Stewart S (2021). A $300 Billion Opportunity: Serving the Emerging Black American Consumer.

[15] Santiago-Torres M, Mull KE, Sullivan BM (2022). Efficacy and utilization of an acceptance and commitment therapy-based smartphone application for smoking cessation among Black adults: secondary analysis of the iCanQuit randomized trial. Addiction.

[16] Bricker JB, Copeland W, Mull KE (2017). Single-arm trial of the second version of an acceptance & commitment therapy smartphone application for smoking cessation. Drug Alcohol Depend.

[17] Iacoviello BM, Steinerman JR, Klein DB (2017). Clickotine, a personalized smartphone app for smoking cessation: initial evaluation. JMIR Mhealth Uhealth.

[18] Enyioha C, Loufman LM, Grewe ME (2023). Black smokers’ preferences for features of a smoking cessation app: qualitative study. JMIR Form Res.

[19] de Bekker-Grob EW, Ryan M, Gerard K (2012). Discrete choice experiments in health economics: a review of the literature. Health Econ.

[20] Soekhai V, de Bekker-Grob EW, Ellis AR, Vass CM (2019). Discrete choice experiments in health economics: past, present and future. Pharmacoeconomics.

[21] Kistler CE, Hess TM, Howard K (2015). Older adults’ preferences for colorectal cancer-screening test attributes and test choice. Patient Prefer Adherence.

[22] Howell M, Howard K, Liamputtong P Handbook of Research Methods in Health Social Sciences.

[23] Ryan M, Scott DA, Reeves C (2001). Eliciting public preferences for healthcare: a systematic review of techniques. Health Technol Assess.

[24] Barrientos-Gutierrez I, Islam F, Cho YJ (2020). Assessing cigarette packaging and labelling policy effects on early adolescents: results from a discrete choice experiment. Tob Control.

[25] Kistler CE, Ranney LM, Sutfin EL (2019). Product attributes important to US adult consumers’ use of electronic nicotine delivery systems: a discrete choice experiment. BMJ Open.

[26] Miller CA, Guidry JPD, Dahman B, Thomson MD (2020). A tale of two diverse Qualtrics samples: information for online survey researchers. Cancer Epidemiol Biomarkers Prev.

[27] Eysenbach G (2004). Improving the quality of Web surveys: the Checklist for Reporting Results of Internet E-Surveys (CHERRIES). J Med Internet Res.

[28] de Bekker-Grob EW, Donkers B, Jonker MF, Stolk EA (2015). Sample size requirements for discrete-choice experiments in healthcare: a practical guide. Patient.

[29] Hauber AB, González JM, Groothuis-Oudshoorn CGM (2016). Statistical methods for the analysis of discrete choice experiments: a report of the ISPOR Conjoint Analysis Good Research Practices Task Force. Value Health.

[30] Reed Johnson F, Lancsar E, Marshall D (2013). Constructing experimental designs for discrete-choice experiments: report of the ISPOR Conjoint Analysis Experimental Design Good Research Practices Task Force. Value Health.

[31] (2017). The CBC system for choice-based conjoint analysis. Version 9. Sawtooth Software.

[32] Jonker MF, Donkers B, de Bekker-Grob E, Stolk EA (2019). Attribute level overlap (and color coding) can reduce task complexity, improve choice consistency, and decrease the dropout rate in discrete choice experiments. Health Econ.

[33] As Predicted.

[34] Brewer LC, Hayes SN, Caron AR (2019). Promoting cardiovascular health and wellness among African-Americans: community participatory approach to design an innovative mobile-health intervention. PLoS ONE.

[35] Nguyen N, Koester KA, Tran C, Ling PM (2024). Desires and needs for quitting both e-cigarettes and cigarettes among young adults: formative qualitative study informing the development of a smartphone intervention for dual tobacco cessation. JMIR Form Res.

[36] Sullivan HW, O’Donoghue AC, Gard Read J, Amoozegar JB, Aikin KJ, Rupert DJ (2018). Testimonials and informational videos on branded prescription drug websites: experimental study to assess influence on consumer knowledge and perceptions. J Med Internet Res.

[37] Newton RL, Carter L, St Romain J, Jerrod T, Griffith DM, Myers V (2019). Development of a mobile phone app to maintain physical activity in African American men: *MobileMen*. Mhealth.

[38] Campbell JA, Walker RJ, Egede LE (2025). Medical mistrust and perceived discrimination as expressions of structural racism: the need for focused research. J Gen Intern Med.

[39] Johnson D, Javed A, Byrnes NJ, Jones AC, Bertsch KN (2025). Influences and implications of medical mistrust on healthcare behaviors in a low health outcomes county in the state of New Jersey. J Community Health.

[40] Jaiswal J, Halkitis PN (2019). Towards a more inclusive and dynamic understanding of medical mistrust informed by science. Behav Med.

[41] Moore D, Mansfield LN, Onsomu EO, Caviness-Ashe N (2022). The role of Black pastors in disseminating COVID-19 vaccination information to Black communities in South Carolina. Int J Environ Res Public Health.

[42] Dennison L, Morrison L, Conway G, Yardley L (2013). Opportunities and challenges for smartphone applications in supporting health behavior change: qualitative study. J Med Internet Res.

[43] Nguyen BT, Brown AL, Jones F (2021). “I’m not going to be a guinea pig:” medical mistrust as a barrier to male contraception for Black American men in Los Angeles, CA. Contraception.

[44] Prather C, Fuller TR, Jeffries WL (2018). Racism, African American women, and their sexual and reproductive health: a review of historical and contemporary evidence and implications for health equity. Health Equity.

[45] Oliver JA, Hallyburton MB, Pacek LR (2018). What do smokers want in a smartphone-based cessation application?. Nicotine Tob Res.

[46] Houston TK, Scarinci IC, Person SD, Greene PG (2005). Patient smoking cessation advice by health care providers: the role of ethnicity, socioeconomic status, and health. Am J Public Health.

[47] El-Hilly AA, Iqbal SS, Ahmed M (2016). Game on? Smoking cessation through the gamification of mHealth: a longitudinal qualitative study. JMIR Serious Games.

[48] Allam A, Kostova Z, Nakamoto K, Schulz PJ (2015). The effect of social support features and gamification on a web-based intervention for rheumatoid arthritis patients: randomized controlled trial. J Med Internet Res.

[49] McClure JB, Hartzler AL, Catz SL (2016). Design considerations for smoking cessation apps: feedback from nicotine dependence treatment providers and smokers. JMIR Mhealth Uhealth.

[50] Xu J, Bricker J, Fu X (2019). Design and development of smoking cessation apps based on smokers’ and providers’ perspectives in China: survey study. JMIR Mhealth Uhealth.

[51] Wang H, Ho AF, Wiener RC, Sambamoorthi U (2021). The association of mobile health applications with self-management behaviors among adults with chronic conditions in the United States. Int J Environ Res Public Health.

[52] Peng W, Kanthawala S, Yuan S, Hussain SA (2016). A qualitative study of user perceptions of mobile health apps. BMC Public Health.

[53] Cheah KJ, Abdul Manaf Z, Fitri Mat Ludin A, Razalli NH, Mohd Mokhtar N, Md Ali SH (2024). Mobile apps for common noncommunicable disease management: systematic search in app stores and evaluation using the Mobile App Rating Scale. JMIR Mhealth Uhealth.

[54] Vilardaga R, Rizo J, Zeng E (2018). User-centered design of learn to quit, a smoking cessation smartphone app for people with serious mental illness. JMIR Serious Games.

[55] Berry KM, Drew JAR, Brady PJ, Widome R (2023). Impact of smoking cessation on household food security. Ann Epidemiol.

[56] Cornelius ME, Wang TW, Jamal A, Loretan CG, Neff LJ (2020). Tobacco product use among adults—United States, 2019. MMWR Morb Mortal Wkly Rep.

[57] Cigarette prices by state. Data Pandas.

[58] Yerger VB, Daniel MR, Malone RE (2005). Taking it to the streets: responses of African American young adults to internal tobacco industry documents. Nicotine Tob Res.

[59] Wells L, Gowda A (2020). A legacy of mistrust: African Americans and the US healthcare system. Proc UCLA Health.

[60] Lewis TJ, Boykin M (2021). We the People: a Black strategy to end the HIV epidemic in the United States of America. J Healthc Sci Humanit.

